# Impact of Built Environments on Body Weight (the Moving to Health Study): Protocol for a Retrospective Longitudinal Observational Study

**DOI:** 10.2196/16787

**Published:** 2020-05-19

**Authors:** Stephen J Mooney, Jennifer F Bobb, Philip M Hurvitz, Jane Anau, Mary Kay Theis, Adam Drewnowski, Anju Aggarwal, Shilpi Gupta, Dori E Rosenberg, Andrea J Cook, Xiao Shi, Paula Lozano, Anne Vernez Moudon, David Arterburn

**Affiliations:** 1 Department of Epidemiology University of Washington Seattle, WA United States; 2 Harborview Injury Prevention & Research Center University of Washington Seattle, WA United States; 3 Kaiser Permanente Washington Health Research Institute Seattle, WA United States; 4 Department of Urban Design and Planning College of Built Environments University of Washington Seattle, WA United States; 5 Center for Public Health Nutrition University of Washington Seattle, WA United States

**Keywords:** electronic health records, obesity, built environment, Washington, geography, longitudinal studies

## Abstract

**Background:**

Studies assessing the impact of built environments on body weight are often limited by modest power to detect residential effects that are small for individuals but may nonetheless comprise large attributable risks.

**Objective:**

We used data extracted from electronic health records to construct a large retrospective cohort of patients. This cohort will be used to explore both the impact of moving between environments and the long-term impact of changing neighborhood environments.

**Methods:**

We identified members with at least 12 months of Kaiser Permanente Washington (KPWA) membership and at least one weight measurement in their records during a period between January 2005 and April 2017 in which they lived in King County, Washington. Information on member demographics, address history, diagnoses, and clinical visits data (including weight) was extracted. This paper describes the characteristics of the adult (aged 18-89 years) cohort constructed from these data.

**Results:**

We identified 229,755 adults representing nearly 1.2 million person-years of follow-up. The mean age at baseline was 45 years, and 58.0% (133,326/229,755) were female. Nearly one-fourth of people (55,150/229,755) moved within King County at least once during the follow-up, representing 84,698 total moves. Members tended to move to new neighborhoods matching their origin neighborhoods on residential density and property values.

**Conclusions:**

Data were available in the KPWA database to construct a very large cohort based in King County, Washington. Future analyses will directly examine associations between neighborhood conditions and longitudinal changes in body weight and diabetes as well as other health conditions.

**International Registered Report Identifier (IRRID):**

DERR1-10.2196/16787

## Introduction

### Background

Residential context—the features of the neighborhoods we live in—affects our health behaviors and well-being [1,2]. Residential environments have been cross-sectionally linked to diet quality, body weight, and prevalence of obesity and obesity-related health conditions [3-7]. However, such study designs have limited causal interpretability owing to challenges isolating the impacts of a single neighborhood exposure and to the threat of reverse causality [8,9]. With a few notable exceptions [10,11], most studies of the impact of changing residential neighborhoods on health operated at the ecological level [12] or leveraged specific one-time changes such as a new transit system [13-15] or supermarket [16,17]. Meanwhile, studies assessing changes in weight among people who moved [18-20] have been limited by modest sample sizes. As neighborhood features often have only modest effects on behavior [21], studies with few participants frequently fail to identify robust and causally interpretable effects of residential environments [22].

### Objectives

The Moving to Health Study, whose design and methods we present here, is using data from Kaiser Permanente Washington (KPWA; formerly Group Health Cooperative) to address this gap [23]. KPWA is a large integrated health insurance and care delivery system in Washington State, serving broad economic strata. By attaching a geographic context to more than a decade of anonymized electronic health records (EHRs) for more than 200,000 adults in King County, Washington (the central county of the Seattle-Tacoma-Bellevue metropolitan statistical area), the study will assess the longitudinal impact of baseline residential built environment, the effect of moving between environments, and the effect of changes in the built environment among those who did not move and on obesity and type 2 diabetes at a heretofore unparalleled scale.

Here, we describe the Moving to Health adult obesity study cohort design, the process of building a longitudinal epidemiologic cohort from health system data, the individual and neighborhood environment characteristics of adults aged 18 years and older in the cohort, and the residential moves that this cohort undertook during 11 years and 4 months of follow-up.

## Methods

### Setting

We constructed a retrospective observational cohort of adults and children in King County, Washington, using data from KPWA merged with publicly available data on the built environment compiled by the Urban Form Lab at the University of Washington. In this paper, we describe the adult cohort; details and analyses regarding the child cohort will be published separately. All study procedures were reviewed in advance and approved by the KPWA institutional review board, approved a waiver of consent, and the Health Insurance Portability and Accountability Act (HIPAA) authorization to identify and enroll study subjects.

KPWA has approximately 700,000 members in Washington, and 36% of these reside in King County. King County includes Seattle and is the most densely populated county in Washington State. KPWA enrollment in King County is similar to the county’s population in terms of income, educational attainment, and representation of racial and ethnic minority groups.

### Data Sources

#### Kaiser Permanente Washington Electronic Health Record

##### Overview

The majority of member care at KPWA is delivered using EHR databases, which also record the majority of clinical outcomes. KPWA medical centers have used the Epic (Epic Systems Corporation) EHR platform since 2005, the first year of our study. The data contained in the EHR data warehouse include the vital indicators of KPWA member health status. For example, biometric data such as heights, weights, and blood pressure values recorded at clinic visits are fully retrievable for analyses, rendering the available patient profiles more detailed than the *insurance claims only* data available from Medicaid, Medicare, or most health plans that contract with independent medical groups or networks of physicians. By combining KPWA EHR data with other extensive databases used in provision of insurance and care (ie, enrollment, outside claims, deaths, costs, outpatient visits, hospitalizations, emergency room care, pharmacy, radiology, and laboratory databases), we can document all medical and surgical care rendered during the period of their enrollment at KPWA for each study participant that was either delivered in (1) KPWA-owned and KPWA-operated medical centers or (2) in KPWA’s contracted network facilities and providers and paid for by the health plan. Specifically, our cohort uses the following data features:

##### Membership

Dates and status of enrollment, types of insurance coverage, and drug coverage plan were used to determine the periods of eligibility as detailed below.

##### Residential Locations

Membership files also contain changes in mailing address, typically the home address (mailing address is confirmed every time a patient contacts KPWA, including clinical visits). We geocoded these home addresses to identify latitude and longitude values for residential locations that can be used to link with spatially referenced data from other sources. A total of 95% of members for whom we attempted to geocode all recorded addresses had at least one address matched successfully. Common sources of inability to geocode included the use of a post office box as a mailing address and a form of address too oblique to be *cleaned* such that the geocoder could find the relevant location. We identified residential relocation (hereafter called moves) by comparing successive address records, such that any change in the patient address that resulted in a different location for the geocoded home address constituted a move. We classified patients for whom we identified a move to another location in the county as *movers* to compare available data for the population whose moves we can analyze to the population as a whole. Geocoding was performed in steps: first, we performed a crude but fast geocode using the SAS (SAS Institute) geocoder with US Census TIGER/Line files to rule out addresses clearly not in King County. Then, to get a more precise home location, we used a composite geocoding approach: we first looked for an exact match in the King County E-911 address points, and then, if no match was identified in the E-911 dataset, we used Esri Business Analyst (ESRI), requiring a *rooftop* match to consider the address successfully geocoded.

##### Demographics

Date of birth, gender, race, and ethnicity are available in the administrative datasets. These data were self-reported by patients as part of routine clinical practice.

##### Clinical Measures

Height and weight are measured by clinical staff and recorded in the EHR during clinical visits. These heights and weights have previously been used extensively for research purposes [4,24]. We excluded weight measurements that clinical expertise indicated were biologically implausible for adults (<70 pounds or ≥700 pounds). Smoking status was self-reported through patient questionnaires deployed during clinical visits.

##### Utilization, Diagnoses, and Procedures

The KPWA EHR includes dates and types of health care utilization for inpatient, emergency department, and outpatient settings. Using the baseline visit and all records dating to the previous 12 months, we constructed an Elixhauser comorbidity score [25,26]. As our baseline was 2005 and all subjects were aged 18 years or older at baseline, we consulted EHR records from as far back as 2004 and for patients as young as 17 years at the time of the visit to construct this score. We also used these records to assess the baseline prevalence of conditions of particular interest, including diabetes, hypertension, dyslipidemia, depression, and anxiety. Codes used to infer the presence of health conditions are available from the authors on request.

#### Measures of Neighborhood Context

**Figure 1:**
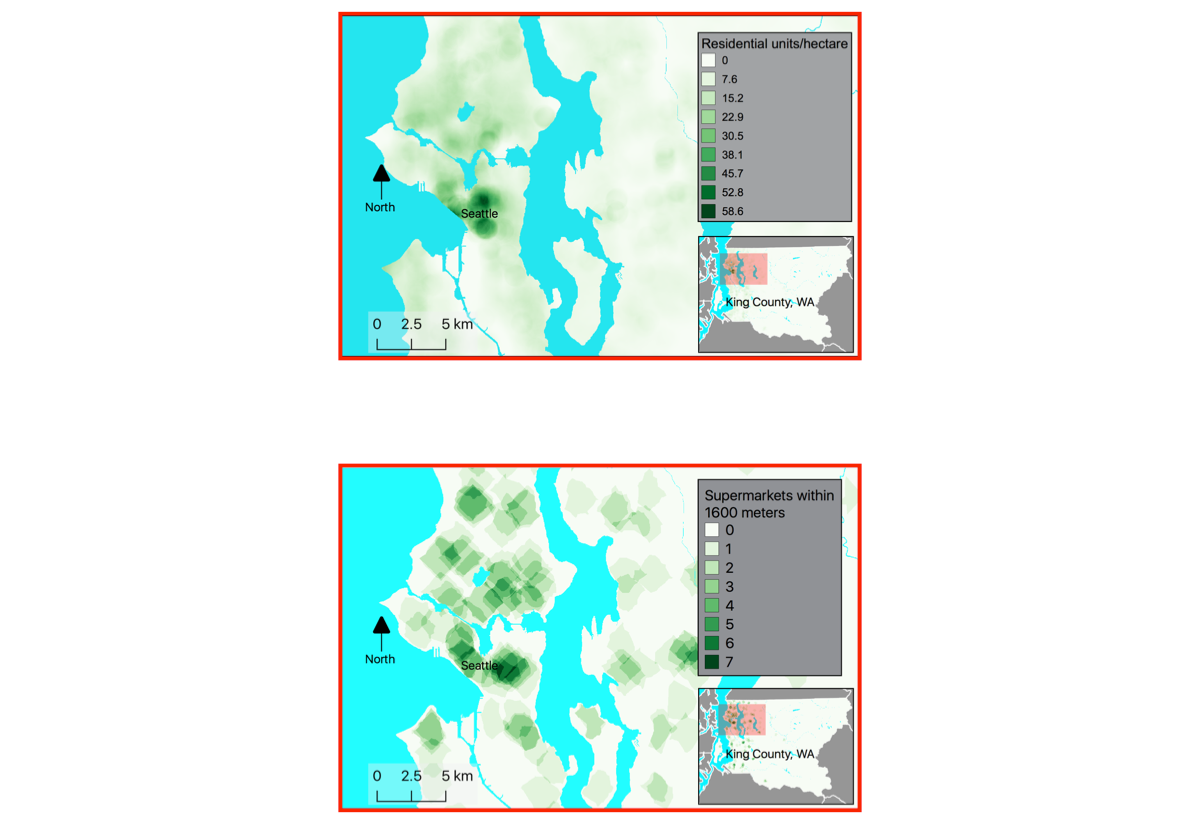
SmartMaps of selected neighborhood measures used in the Moving to Health Cohort, 2005 to 2017. The top panel shows residential density in Western King County within 800 m (inset map of greater King County) in 2005. The bottom panel supermarket count within 1600 m in the same area in 2008.

As of December 2019, we have constructed six neighborhood environment measures (Table 1) and anticipate constructing more. These measures are drawn from publicly available geographic information systems (GIS) data layers and were selected to assess aspects of neighborhoods thought to influence physical activity behaviors and weight trajectory. Obtaining multiple GIS-based environmental measures for hundreds of thousands of point locations is challenging; to accomplish this, for each variable of interest, we first constructed SmartMaps [27], which are spatially continuous rasterized surfaces, where each raster cell contains the average value of the environmental feature of interest within a predetermined distance (Figure 1). The maps allow efficient estimation of environmental characteristics for large numbers of point locations. We used each SmartMap to assign the selected neighborhood measure to each subject home location at baseline and multiple follow-ups, based on historical GIS data temporally matched with the EHR. This approach avoids typical GIS workflows that require computing each environmental measure for each individual geocoded location. We used radial buffers rather than network buffers for most SmartMaps to minimize computational costs. An additional advantage of the SmartMap approach is that SmartMaps can be constructed by team members outside of KPWA without the need for HIPAA-protected home addresses. SmartMaps were developed using PostgreSQL, PostGIS, and R (R Foundation).

**Table 1:** Selected neighborhood built environment variables in the Moving to Health cohort study.

Domain and variable^a^		Data source	Median values for 1600 m buffer at baseline (first quartile, third quartile)	Years of data available	Radial buffer distance (m)
**Neighborhood composition**					
	Residential density, units/hectare	King County Assessor’s office	9 (6, 15)	2005-2017	800, 1600
	Population density, residents/hectare	American Community Survey	21 (14, 31)	2005-2017	800, 1600
	Property value per residential unit (US $), 2017	King County Assessor’s office	282,949 (21,543; 373 470)	2005-2017	800, 1600
**Transportation systems**					
	Street intersection density, intersections/hectare	TIGER/Line files	0.6 (0.5, 0.8)	2010-2018	800, 1600
**Food environment**					
	Supermarket count	PHSKC^b^/UFL^c^	1 (0, 2)	2008, 2012, 2015	1600, 5000
	Fast food retailer count	PHSKC/UFL	2 (0, 6)	2008, 2012, 2015	1600, 5000

^a^These variables have been constructed. Additional variables are planned as described in the manuscript text, and new variables can be added as data become available.

^b^PHSKC: Seattle/King County Public Health department.

^c^UFL: University of Washington Urban Form Lab.

We have constructed measures covering the following domains of neighborhood conditions; however, a key feature of our cohort design is that other measures of the built environment can be easily added in the future as the data become available:

#### Neighborhood Composition

The physical and social composition of a neighborhood may influence walkable access to retail and daily routine destinations, perceptions of the safety of outdoor physical activity, and other weight-relevant behavioral health norms. Our neighborhood composition measures included residential density (housing units/land area) [28-30] and population density (residents/land area) [6,31,32] to capture the intensity of neighborhood development and related mix of land uses, as well as residential property values as a dimension of neighborhood socioeconomic status [5]. We will develop a measure of employment density for use with this cohort.

#### Transportation Infrastructure

Transportation infrastructure affects a resident’s ability to choose active transportation options, which, in turn, may prevent obesity. Street intersection density, a measure of walking connectivity, has been found to be negatively associated with obesity, albeit inconsistently [33,34]. Similarly, access to sidewalks and trails is also thought to encourage walking and prevent obesity, although findings focused on walking infrastructure have also been inconsistent [35-37]. We have measured street intersection density from King County GIS data and will measure trail density using King County GIS data and transit ridership per bus stop as reported by King County Metro, which operates the bus system within the county.

#### Food Environment

The food environment has been strongly correlated with obesity, but questions remain as to whether the relationship is causal [6,38,39]. Measures of the food environment for our cohort included densities of supermarkets and fast food restaurants as reported by King County Public Health and geoprocessed by the University of Washington Urban Form Lab [40], and we will construct a similar measure of convenience stores. As most King County residents drive to shop for food [41], the SmartMaps for food environment measures used network buffers to account for road network impacts on driving distances.

#### Recreational and Fitness Environments

Neighborhood parks are thought to encourage physical activity that prevents unhealthy weight gain [42,43]. We will compute the percent of land area dedicated to parks as reported by King County and local municipalities and compiled by the University of Washington Urban Form Lab [42]. Future analyses may also incorporate gyms, exercise studios, swimming pools, and other venues for recreational activity.

### Identifying a Cohort From Electronic Health Record Data

**Figure 2:**
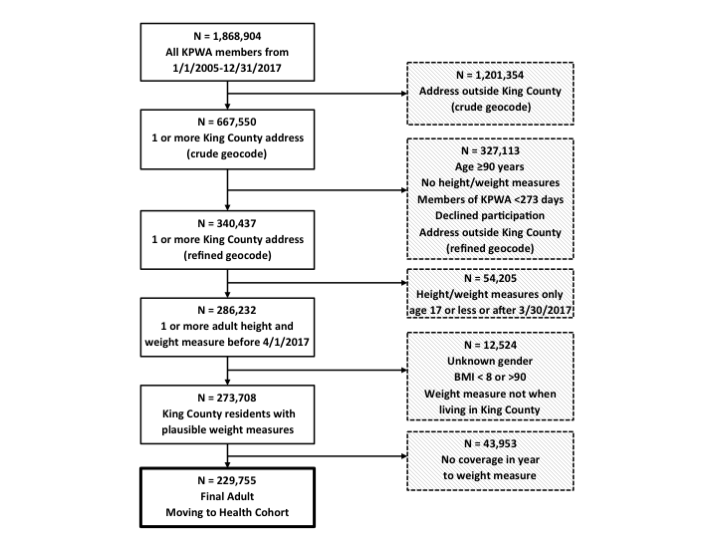
Flow diagram showing selection from the Kaiser Permanente Washington membership to the Moving to Health Cohort.

To construct the study cohort, we initially identified KPWA members aged 18 to 89 years between January 1, 2005, and December 31, 2017, whose home addresses were successfully geocoded to a King County location and for whom height and weight data were available. We required a successful geocode because our goal was to assess the impacts of residential location. We excluded members older than 89 years owing to concerns that older age could be personally identifying. We later determined that an EHR system change rendered address changes after April 30, 2017 inconsistent and limited our data to records of visits before May 1, 2017. We included KPWA members who had a recorded weight measure while they were a resident of King County, Washington, after having been a KPWA member for at least 1 year to help ensure we had sufficient data to estimate the prevalence of comorbid health conditions before their weight measurement. Figure 2 is a flow diagram describing the identification of this cohort.

### Follow-Up and Outcomes

We defined the first eligible weight measure of an individual in the cohort to be their *baseline* measure. We considered a member to be followed at each clinic visit after the baseline visit and censored before the end of follow-up if he or she moved out of King County or was not a member of KPWA for at least 13 months. Once censored, individuals did not rejoin the cohort even if they became KPWA members again. We did not censor women during pregnancy. This will allow us to conduct analyses incorporating pregnancy weight change; however, we anticipate that analyses not focused on pregnancy will need to handle pregnancy episodes appropriately.

**Figure 3:**
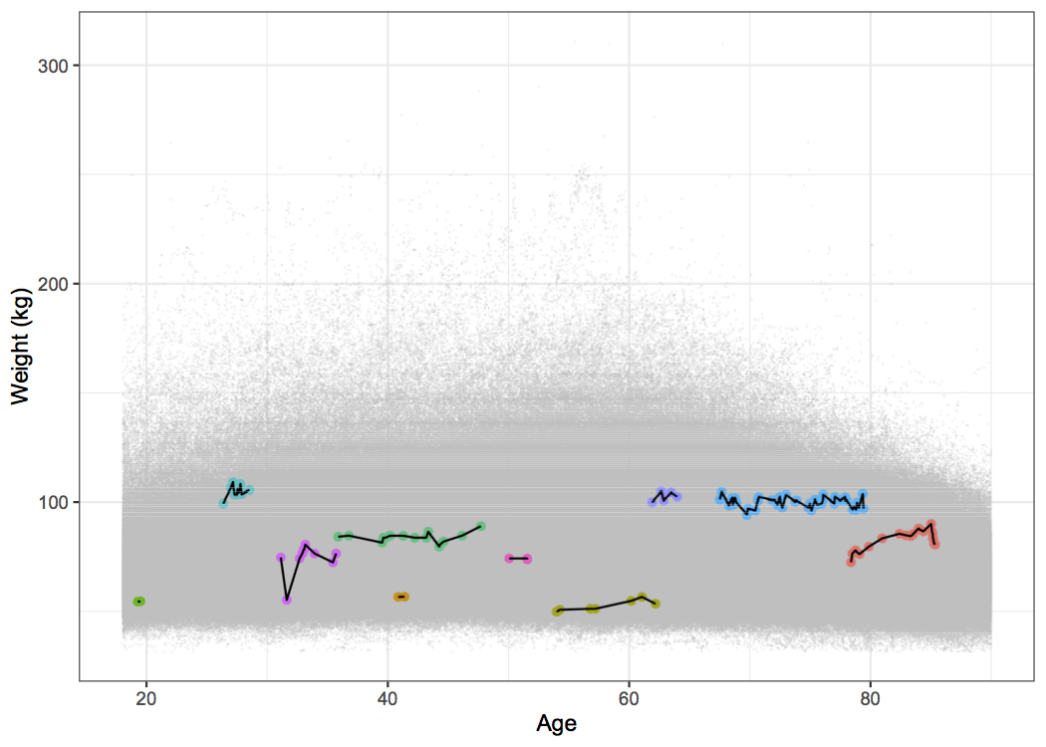
Weight values recorded in the Moving to Health adult cohort, 2005 to 2017, with selected individual weight trajectories highlighted to demonstrate the range of within-subject follow-up, variability, and weight trajectory over time.

The primary outcome of our future analyses will be weight change over time. We intend to focus on weight change rather than BMI change to minimize artifacts that could arise because of the height measurement error in this cohort of adults whose height change should be minimal. Figure 3 is a plot of weight measurements over time, with trajectories of selected study subjects highlighted as examples. There is substantial variability in weight trajectory, follow-up, and within-subject variability over time. Additional analyses will examine changes in glycemic control among patients with type 2 diabetes, as measured by the serum glycosylated hemoglobin test; these outcomes will be described in future manuscripts.

### Analyses

The analyses for this cohort description manuscript focused on baseline characteristics of the study cohort, comparison of movers with nonmovers to the full cohort, and exploration of the characteristics of residential moves undertaken by cohort members. All analyses were descriptive and conducted in R for Windows version 3.5.2 (Vienna, Austria).

## Results

### Exclusions

The records of 4,208,674 clinic visits that included a weight assessment among 286,232 unique adults met initial inclusion criteria. After applying the exclusion and censoring criteria as depicted in Figure 2, 3,061,603 visits by 229,755 adults remained. Most exclusions (43,953/229,755, 19.1%) of the adults identified in the initial data extraction) were subjects for whom a baseline weight measure could not be identified because the EHR included no weight measure during a time window in which the subject had been a KPWA member for the prior year.

### Population Characteristics

The final study population was a broad cross-section of King County adults (Table 2). Nearly 58.0% (133,326/229,755) of the study population was female, the mean age was 45.0 years, and approximately 59.5% (136,793/229,755) reported non-Hispanic white race/ethnicity. The mean BMI at baseline was 27.7 kg/m^2^, and about 70.1% (161,246/229,755) of the participants were in the 18.5 to 30 kg/m^2^ BMI range typically associated with the lowest mortality risk. The IQR for BMI at baseline was 23.2 to 30.7.

**Table 2:** Baseline characteristics of participants in Moving to Health Cohort Study, King County, Washington, 2005 to 2017.

Characteristic		Total (N=229,755)	Moved within county (n=55,152)	Never moved within county (n=174,603)
Years of follow-up, mean (SD)		5.0 (3.7)	6.1 (3.5)	4.6 (3.7)
**Year of cohort entry, n (%)**				
	2005-2007	101,543 (44.2)	26,501 (48.1)	75,042 (43.0)
	2008-2010	38,487 (16.8)	11,308 (20.5)	27,179 (15.6)
	2011-2013	49,410 (21.5)	11,692 (21.2)	37,718 (21.6)
	2014-2017	40,315 (17.5)	5651 (10.2)	34,664 (19.9)
Age in years at cohort entry, mean (SD)		45.0 (17.3)	41.5 (17.1)	46.2 (17.2)
**Age categories (years), n (%)**				
	18-29	55,624 (24.2)	17,519 (31.8)	38,105 (21.8)
	30-44	62,861 (27.4)	17,504 (31.7)	45,357 (26.0)
	45-54	42,030 (18.3)	7991 (14.5)	34,039 (19.5)
	55-64	38,212 (16.6)	5940 (10.8)	32,272 (18.5)
	65-89	31,007 (13.5)	6194 (11.2)	24,813 (14.2)
**Gender, n (%)**				
	Male	96,429 (42.0)	21,658 (39.3)	74,771 (42.8)
**Race/ethnicity, n (%)**				
	Asian	27,573 (12.0)	6496 (11.8)	21,077 (12.1)
	Black	13,420 (5.8)	4096 (7.4)	9324 (5.3)
	Hawai’ian/Pacific Islander	2278 (1.0)	694 (1.3)	1584 (0.9)
	Hispanic	11,275 (4.9)	3127 (5.7)	8148 (4.7)
	Native American/Alaskan Native	2585 (1.1)	671 (1.2)	1914 (1.1)
	Other	2797 (1.2)	726 (1.3)	2071 (1.2)
	Unknown	33,034 (14.4)	6745 (12.2)	26,289 (15.1)
	Non-Hispanic white	136,793 (59.5)	32,597 (59.1)	104,196 (59.7)
Height (m), mean (SD)^a^		1.69 (0.1)	1.69 (0.1)	1.69 (0.1)
Weight (kg), mean (SD)		79.3 (21.0)	78.6 (21.1)	79.5 (21.0)
BMI (kg/m^2^), mean (SD)		27.7 (6.4)	27.5 (6.5)	27.8 (6.4)
**BMI categories (kg/m^2^), n (%)^a^**				
	<18.5	3399 (1.5)	873 (1.6)	2526 (1.5)
	18.5-25.0	85,572 (37.4)	22,114 (40.2)	63,458 (36.6)
	25.0-29.9	75,674 (33.1)	17,325 (31.5)	58,349 (33.6)
	30.0-34.9	36,745 (16.1)	8266 (15.0)	28,479 (16.4)
	≥35.0	27,056 (11.8)	6395 (11.6)	20,661 (11.9)
**Weight measurements**				
	Number of BMI measures, mean (SD)	13.3 (17.8)	17.0 (19.4)	12.2 (17.2)
	Any BMI measures 1+ years apart, n (%)	154,040 (67.0)	46,868 (85.0)	107,172 (61.4)
	Any BMI measures 3+ years apart, n (%)	103,314 (45.0)	33,847 (61.4)	69,467 (39.8)
	Any BMI measures 5+ years apart, n (%)	72,726 (31.7)	23,798 (43.2)	48,928 (28.0)
	Any BMI measures 9+ years apart, n (%)	37,612 (16.4)	10,971 (19.9)	26,641 (15.3)
Elixhauser score, mean (SD)		0.7 (1.2)	0.7 (1.1)	0.7 (1.2)
**Comorbidities prior to baseline, n (%)**				
	Diabetes	13,345 (5.8)	2786 (5.1)	10,559 (6.0)
	Hypertension	30,182 (13.1)	5907 (10.7)	24,275 (13.9)
	Dyslipidemia	17,964 (7.8)	3165 (5.7)	14,799 (8.5)
	Depression	23,385 (10.2)	6166 (11.2)	17,219 (9.9)
	Anxiety	18,636 (8.1)	5016 (9.1)	13,620 (7.8)
**Smoking status, n (%)^b^**				
	Current	23,920 (13.2)	6237 (14.4)	17,683 (12.8)
	Former	35,915 (19.7)	8198 (19.0)	27,717 (20.0)
	Never	120,654 (66.3)	28,511 (66.0)	92,143 (66.5)
	Did not respond	1362 (0.7)	265 (0.6)	1097 (0.8)
Property value per unit at home address, 2017 (US $), mean (SD)^c^		354,464 (265,517)	313,455 (263,759)	366,932 (264,795)

^a^Modal height missing from 0.5% of the cohort.

^b^Smoking status missing from 20.9% of the cohort who never received survey.

^c^Property values at home address missing from 9.8% of the cohort.

### Follow-Up

The baseline visit for approximately 44.1% (101,543/229,755) of the final analytic cohort was in the first 3 years of study enrollment, between January 1, 2005, and December 31, 2007. The mean follow-up was slightly less than 5 years, and follow-up ranged from 1 day to 12 years and 118 days, 3 days shy of the full follow-up period. Weight measures at least 1 year apart were available for 67.0% (154,040/229,755) of subjects, measures at least 5 years apart were available for 31.6% (72,726/229,755) of subjects, and measures at least 9 years apart were available for 16.3% (37,612/229,755) of subjects. In addition, 43.9% (101,053/229,755) of subjects were still enrolled at the end of study follow-up; the most common (87,116/229,755, 37.9%) reason for censoring was that the subject disenrolled from KPWA for at least 13 months.

### Moves

**Figure 4:**
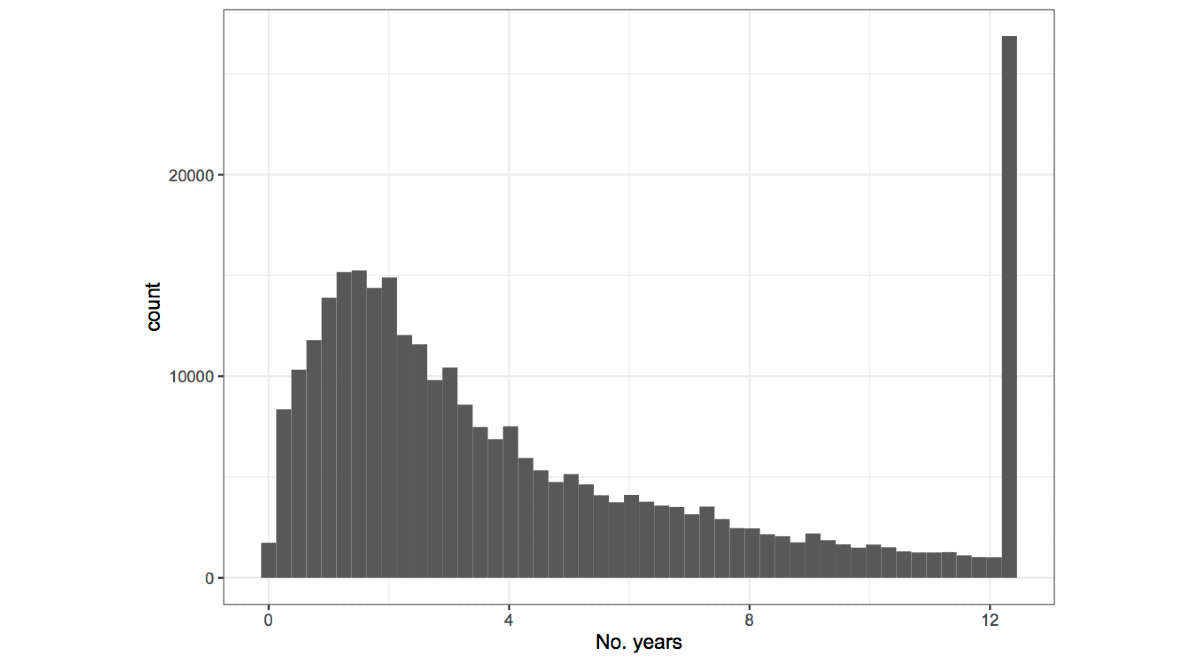
Histogram of location-specific follow-up (residential tenure) in the Moving to Health cohort, 2005 to 2017. The peak around 13 years corresponds to people who were enrolled throughout the full study period without moving.

Approximately 24.0% (55,152/229,755) of the cohort moved at least once during follow-up. Movers were a somewhat younger subcohort (mean age 41.5 years among movers compared with 45.0 overall) and tended toward longer follow-up (54% followed for 5 years or more compared with 39% overall). This may be because those who remained a member with KPWA for longer had a greater probability of their membership time overlapping with a move. In addition, 67.8% (37,388/55,152) of movers moved only once during the follow-up. Figure 4 is a histogram of residential tenure at each address tracked in the study.

**Figure 5:**
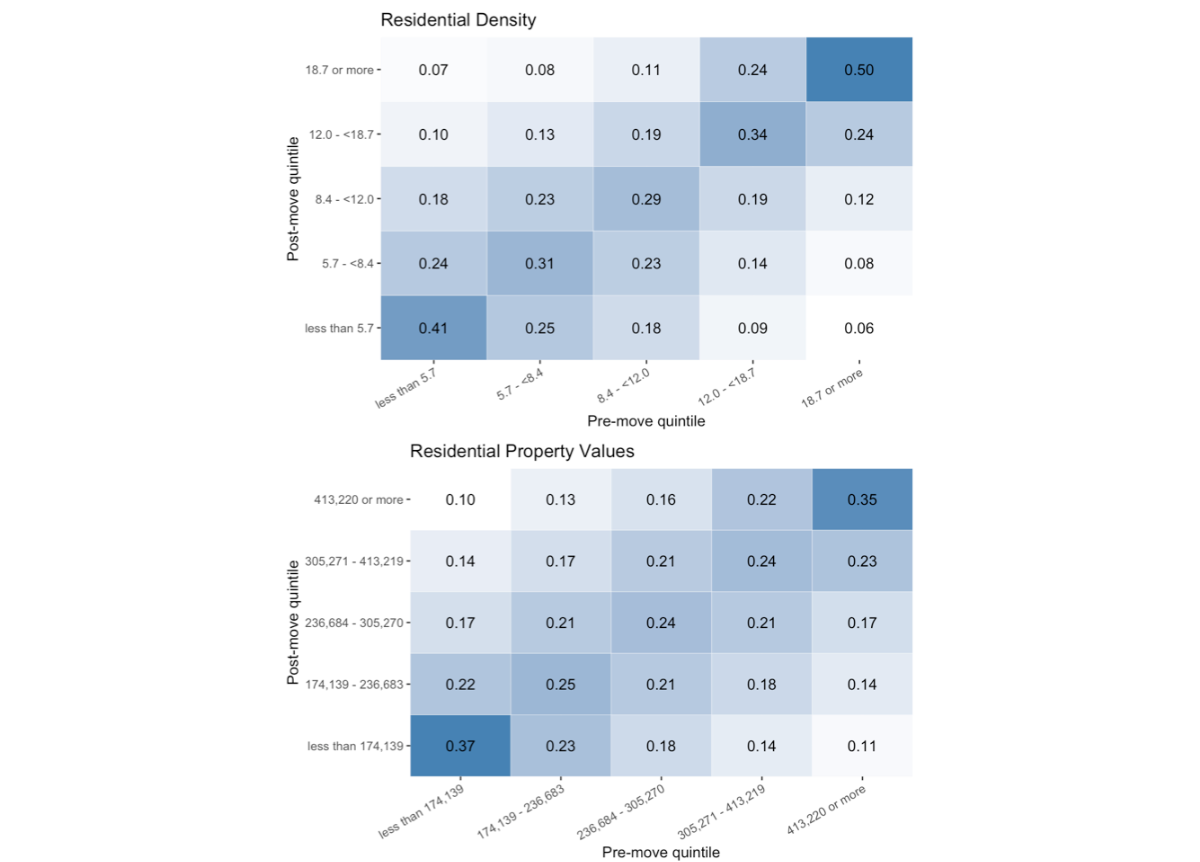
Heat maps showing quintiles of neighborhood residential density and property value within 800 m across moves among persons in the Moving to Health cohort, 2005 to 2017. Numbers in grid cells indicate the proportion of those in the premove quintile whose move destination was in the associated postmove quintile. For example, the top right corner of the top panel indicates that 50% (9519/19,107) of those living in locations where residential densities were 18.7 units/hectare or more before a move moved to locations with residential densities of 18.7 units/hectare or more.

In total, the 55,152 movers made 84,698 moves (Table 3). A total of 45.9% (38,911/84,698) of these moves were less than 5 km in distance, and destinations had residential densities and property values more like origins than would be expected by chance (χ^2^ test *P*<.001). For example, although only 19.8% (16,803/84,698) of moves were initiated from residential locations with densities of 18.7 units/hectare (roughly that of a 1920’s era *streetcar suburb* neighborhood) or more, 53.5% (8962/16,803) of those moves were to destinations that also had residential densities of 18.8 units/hectare or above (Figure 5, top panel).

**Table 3:** Selected characteristics of the 84,698 residential moves within King County, Washington, occurring during Moving to Health Cohort follow-up, 2005 to 2017.

Characteristic		Change
**Order of move, n (%)**		
	First move for this member	55,152 (65.1)
	Second move for this member	17,764 (21.0)
	Third or more move for this member	11,782 (13.9)
**Year of move, n (%)**		
	2005-2007	16,443 (19.4)
	2008-2010	20,118 (23.8)
	2011-2013	23,365 (27.6)
	2014-2017	24,772 (29.2)
**Distance between residential locations in the move (km), n (%)**		
	<1	11,003 (13.0)
	1-4.9	27,908 (33.0)
	≥5.0	45,787 (54.1)
**Change in selected neighborhood characteristics, median (first quartile, third quartile)**		
	Residential density within 800 m, housing units/hectare	0.1 (−4.1, 4.2)
	Population density within 800 m, population/hectare	−0.5 (−9.4, 7.5)
	Street intersection density within 800 m, intersections/hectare	0 (−.19, .15)
	Mean residential property value within 800 m ($), 2017	−9173 (−113 805, 8 9 537)
	Supermarket count within 1600 m	0 (−1, 1)
	Fast food restaurant count within 1600 m	0 (−3, 2)

## Discussion

### Principal Findings

In this population-based, retrospective cohort constructed from KPWA medical records, we have identified 229,755 adults aged 18 to 89 years who lived in King County, Washington, who were continuously enrolled in KPWA for at least 1 year, and for whom at least one weight measure is available for analysis. Of these adults, an average of about 5 years of follow-up was available, and 55,152 moved within the county at least once.

To the best of our knowledge, this is the first large-scale EHR-based cohort developed to assess the impact of residential moves on the health of adults [44]. However, there is prior work assessing neighborhood influences on BMI change in children using EHR data [45], and there is a substantial literature on the reasons that people change the residential location and the process by which movers select residential locations [23,46-48]. Our finding that nearly half of our recorded moves were within 5 km of the initial residential location is consistent with prior findings that moves in Western Washington and elsewhere tend to be within corridors or neighborhoods [49,50]. As short distance moves imply limited changes to neighborhood built environments, substantial statistical power is needed to assess the impacts of moves.

### Strengths and Limitations

Indeed, the sample size and considerable follow-up time available are key strengths of this cohort [10,11]. Individual health impacts of built environments are likely to be small in general, but because many people are affected by the same characteristics, impacts that may be small at the individual level can still have large population impacts [1]. Another key strength of our design is our use of EHR cohorts for population inferences [51]; our design may act as a template for future similar studies in other populations in other geographic contexts. The sample size is large for examining health outcomes such as obesity, type 2 diabetes, and hypertension, and data on health outcomes are comprehensive in that they include all diagnoses and treatments paid for by Kaiser Permanente insurance during the study period. More generally, our work was possible only because of a foresighted health system decision to treat residential address as patient data to be recorded longitudinally rather than contact information to be updated without maintaining the old value.

Studies using our cohort will also be subject to several limitations. First, this is an EHR cohort, and the research team is not interacting with study subjects directly, which precludes collecting some data that may be readily available in more conventional cohort designs. For example, there are no available measures of the behaviors through which exposure to neighborhood environments might affect weight change, such as physical activity or diet. Second, because the data were not initially collected for research purposes, some potentially relevant covariates are missing (eg, race/ethnicity, particularly in the early years of the cohort), and we cannot verify whether those data are missing at random. Third, weight change, which captures not only changes in fat mass but also changes in lean mass, can be challenging to interpret as an indicator of health [52]. Fourth, our cohort excludes members who listed a post office box address or whose address otherwise could not be geocoded, who may be different from other members. Fifth, residential address recorded in the EHR does not fully capture a subject’s environment, both because residential environment is only a subset of environment encountered and because address in the dataset may only partially reflect the true home location of some members, such as students attending college. Finally, although King County is large and geographically diverse and our cohort demographics resemble those of the county as a whole, county residents are wealthy relative to the rest of Washington State, and the region has fewer African American and Hispanic residents than the country as a whole.

### Conclusions

In conclusion, the Moving to Health Cohort is a very large, EHR-based cohort that offers novel potential for identifying neighborhood effects on obesity and obesity-related conditions.

## Abbreviations

EHR: electronic health record
GIS: geographic information systems
HIPAA: Health Insurance Portability and Accountability Act
KPWA: Kaiser Permanente Washington
